# Measurement of the production cross section of a W boson in association with two b jets in pp collisions at $\sqrt{s} = 8{\,\mathrm{{TeV}}}$

**DOI:** 10.1140/epjc/s10052-016-4573-z

**Published:** 2017-02-11

**Authors:** V. Khachatryan, A. M. Sirunyan, A. Tumasyan, W. Adam, E. Asilar, T. Bergauer, J. Brandstetter, E. Brondolin, M. Dragicevic, J. Erö, M. Flechl, M. Friedl, R. Frühwirth, V. M. Ghete, C. Hartl, N. Hörmann, J. Hrubec, M. Jeitler, A. König, I. Krätschmer, D. Liko, T. Matsushita, I. Mikulec, D. Rabady, N. Rad, B. Rahbaran, H. Rohringer, J. Schieck, J. Strauss, W. Treberer-Treberspurg, W. Waltenberger, C. -E. Wulz, V. Mossolov, N. Shumeiko, J. Suarez Gonzalez, S. Alderweireldt, E. A. De Wolf, X. Janssen, J. Lauwers, M. Van De Klundert, H. Van Haevermaet, P. Van Mechelen, N. Van Remortel, A. Van Spilbeeck, S. Abu Zeid, F. Blekman, J. D’Hondt, N. Daci, I. De Bruyn, K. Deroover, N. Heracleous, S. Lowette, S. Moortgat, L. Moreels, A. Olbrechts, Q. Python, S. Tavernier, W. Van Doninck, P. Van Mulders, I. Van Parijs, H. Brun, C. Caillol, B. Clerbaux, G. De Lentdecker, H. Delannoy, G. Fasanella, L. Favart, R. Goldouzian, A. Grebenyuk, G. Karapostoli, T. Lenzi, A. Léonard, J. Luetic, T. Maerschalk, A. Marinov, A. Randle-conde, T. Seva, C. Vander Velde, P. Vanlaer, R. Yonamine, F. Zenoni, F. Zhang, A. Cimmino, T. Cornelis, D. Dobur, A. Fagot, G. Garcia, M. Gul, D. Poyraz, S. Salva, R. Schöfbeck, M. Tytgat, W. Van Driessche, E. Yazgan, N. Zaganidis, H. Bakhshiansohi, C. Beluffi, O. Bondu, S. Brochet, G. Bruno, A. Caudron, S. De Visscher, C. Delaere, M. Delcourt, L. Forthomme, B. Francois, A. Giammanco, A. Jafari, P. Jez, M. Komm, V. Lemaitre, A. Magitteri, A. Mertens, M. Musich, C. Nuttens, K. Piotrzkowski, L. Quertenmont, M. Selvaggi, M. Vidal Marono, S. Wertz, N. Beliy, W. L. Aldá Júnior, F. L. Alves, G. A. Alves, L. Brito, C. Hensel, A. Moraes, M. E. Pol, P. Rebello Teles, E. Belchior Batista Das Chagas, W. Carvalho, J. Chinellato, A. Custódio, E. M. Da Costa, G. G. Da Silveira, D. De Jesus Damiao, C. De Oliveira Martins, S. Fonseca De Souza, L. M. Huertas Guativa, H. Malbouisson, D. Matos Figueiredo, C. Mora Herrera, L. Mundim, H. Nogima, W. L. Prado Da Silva, A. Santoro, A. Sznajder, E. J. Tonelli Manganote, A. Vilela Pereira, S. Ahuja, C. A. Bernardes, S. Dogra, T. R. Fernandez Perez Tomei, E. M. Gregores, P. G. Mercadante, C. S. Moon, S. F. Novaes, Sandra S. Padula, D. Romero Abad, J. C. Ruiz Vargas, A. Aleksandrov, R. Hadjiiska, P. Iaydjiev, M. Rodozov, S. Stoykova, G. Sultanov, M. Vutova, A. Dimitrov, I. Glushkov, L. Litov, B. Pavlov, P. Petkov, W. Fang, M. Ahmad, J. G. Bian, G. M. Chen, H. S. Chen, M. Chen, Y. Chen, T. Cheng, C. H. Jiang, D. Leggat, Z. Liu, F. Romeo, S. M. Shaheen, A. Spiezia, J. Tao, C. Wang, Z. Wang, H. Zhang, J. Zhao, Y. Ban, G. Chen, Q. Li, S. Liu, Y. Mao, S. J. Qian, D. Wang, Z. Xu, C. Avila, A. Cabrera, L. F. Chaparro Sierra, C. Florez, J. P. Gomez, C. F. González Hernández, J. D. Ruiz Alvarez, J. C. Sanabria, N. Godinovic, D. Lelas, I. Puljak, P. M. Ribeiro Cipriano, Z. Antunovic, M. Kovac, V. Brigljevic, D. Ferencek, K. Kadija, S. Micanovic, L. Sudic, T. Susa, A. Attikis, G. Mavromanolakis, J. Mousa, C. Nicolaou, F. Ptochos, P. A. Razis, H. Rykaczewski, M. Finger, M. Finger Jr., E. Carrera Jarrin, Y. Assran, T. Elkafrawy, A. Mahrous, B. Calpas, M. Kadastik, M. Murumaa, L. Perrini, M. Raidal, A. Tiko, C. Veelken, P. Eerola, J. Pekkanen, M. Voutilainen, J. Härkönen, V. Karimäki, R. Kinnunen, T. Lampén, K. Lassila-Perini, S. Lehti, T. Lindén, P. Luukka, T. Peltola, J. Tuominiemi, E. Tuovinen, L. Wendland, J. Talvitie, T. Tuuva, M. Besancon, F. Couderc, M. Dejardin, D. Denegri, B. Fabbro, J. L. Faure, C. Favaro, F. Ferri, S. Ganjour, S. Ghosh, A. Givernaud, P. Gras, G. Hamel de Monchenault, P. Jarry, I. Kucher, E. Locci, M. Machet, J. Malcles, J. Rander, A. Rosowsky, M. Titov, A. Zghiche, A. Abdulsalam, I. Antropov, S. Baffioni, F. Beaudette, P. Busson, L. Cadamuro, E. Chapon, C. Charlot, O. Davignon, R. Granier de Cassagnac, M. Jo, S. Lisniak, P. Miné, M. Nguyen, C. Ochando, G. Ortona, P. Paganini, P. Pigard, S. Regnard, R. Salerno, Y. Sirois, T. Strebler, Y. Yilmaz, A. Zabi, J. -L. Agram, J. Andrea, A. Aubin, D. Bloch, J. -M. Brom, M. Buttignol, E. C. Chabert, N. Chanon, C. Collard, E. Conte, X. Coubez, J. -C. Fontaine, D. Gelé, U. Goerlach, A. -C. Le Bihan, J. A. Merlin, K. Skovpen, P. Van Hove, S. Gadrat, S. Beauceron, C. Bernet, G. Boudoul, E. Bouvier, C. A. Carrillo Montoya, R. Chierici, D. Contardo, B. Courbon, P. Depasse, H. El Mamouni, J. Fan, J. Fay, S. Gascon, M. Gouzevitch, G. Grenier, B. Ille, F. Lagarde, I. B. Laktineh, M. Lethuillier, L. Mirabito, A. L. Pequegnot, S. Perries, A. Popov, D. Sabes, V. Sordini, M. Vander Donckt, P. Verdier, S. Viret, T. Toriashvili, I. Bagaturia, C. Autermann, S. Beranek, L. Feld, A. Heister, M. K. Kiesel, K. Klein, M. Lipinski, A. Ostapchuk, M. Preuten, F. Raupach, S. Schael, C. Schomakers, J. F. Schulte, J. Schulz, T. Verlage, H. Weber, V. Zhukov, M. Brodski, E. Dietz-Laursonn, D. Duchardt, M. Endres, M. Erdmann, S. Erdweg, T. Esch, R. Fischer, A. Güth, M. Hamer, T. Hebbeker, C. Heidemann, K. Hoepfner, S. Knutzen, M. Merschmeyer, A. Meyer, P. Millet, S. Mukherjee, M. Olschewski, K. Padeken, T. Pook, M. Radziej, H. Reithler, M. Rieger, F. Scheuch, L. Sonnenschein, D. Teyssier, S. Thüer, V. Cherepanov, G. Flügge, W. Haj Ahmad, F. Hoehle, B. Kargoll, T. Kress, A. Künsken, J. Lingemann, A. Nehrkorn, A. Nowack, I. M. Nugent, C. Pistone, O. Pooth, A. Stahl, M. Aldaya Martin, C. Asawatangtrakuldee, K. Beernaert, O. Behnke, U. Behrens, A. A. Bin Anuar, K. Borras, A. Campbell, P. Connor, C. Contreras-Campana, F. Costanza, C. Diez Pardos, G. Dolinska, G. Eckerlin, D. Eckstein, E. Eren, E. Gallo, J. Garay Garcia, A. Geiser, A. Gizhko, J. M. Grados Luyando, P. Gunnellini, A. Harb, J. Hauk, M. Hempel, H. Jung, A. Kalogeropoulos, O. Karacheban, M. Kasemann, J. Keaveney, J. Kieseler, C. Kleinwort, I. Korol, D. Krücker, W. Lange, A. Lelek, J. Leonard, K. Lipka, A. Lobanov, W. Lohmann, R. Mankel, I. -A. Melzer-Pellmann, A. B. Meyer, G. Mittag, J. Mnich, A. Mussgiller, E. Ntomari, D. Pitzl, R. Placakyte, A. Raspereza, B. Roland, M. Ö. Sahin, P. Saxena, T. Schoerner-Sadenius, C. Seitz, S. Spannagel, N. Stefaniuk, K. D. Trippkewitz, G. P. Van Onsem, R. Walsh, C. Wissing, V. Blobel, M. Centis Vignali, A. R. Draeger, T. Dreyer, E. Garutti, K. Goebel, D. Gonzalez, J. Haller, M. Hoffmann, A. Junkes, R. Klanner, R. Kogler, N. Kovalchuk, T. Lapsien, T. Lenz, I. Marchesini, D. Marconi, M. Meyer, M. Niedziela, D. Nowatschin, J. Ott, F. Pantaleo, T. Peiffer, A. Perieanu, J. Poehlsen, C. Sander, C. Scharf, P. Schleper, A. Schmidt, S. Schumann, J. Schwandt, H. Stadie, G. Steinbrück, F. M. Stober, M. Stöver, H. Tholen, D. Troendle, E. Usai, L. Vanelderen, A. Vanhoefer, B. Vormwald, C. Barth, C. Baus, J. Berger, E. Butz, T. Chwalek, F. Colombo, W. De Boer, A. Dierlamm, S. Fink, R. Friese, M. Giffels, A. Gilbert, P. Goldenzweig, D. Haitz, F. Hartmann, S. M. Heindl, U. Husemann, I. Katkov, P. Lobelle Pardo, B. Maier, H. Mildner, M. U. Mozer, T. Müller, Th. Müller, M. Plagge, G. Quast, K. Rabbertz, S. Röcker, F. Roscher, M. Schröder, I. Shvetsov, G. Sieber, H. J. Simonis, R. Ulrich, J. Wagner-Kuhr, S. Wayand, M. Weber, T. Weiler, S. Williamson, C. Wöhrmann, R. Wolf, G. Anagnostou, G. Daskalakis, T. Geralis, V. A. Giakoumopoulou, A. Kyriakis, D. Loukas, I. Topsis-Giotis, A. Agapitos, S. Kesisoglou, A. Panagiotou, N. Saoulidou, E. Tziaferi, I. Evangelou, G. Flouris, C. Foudas, P. Kokkas, N. Loukas, N. Manthos, I. Papadopoulos, E. Paradas, N. Filipovic, G. Bencze, C. Hajdu, P. Hidas, D. Horvath, F. Sikler, V. Veszpremi, G. Vesztergombi, A. J. Zsigmond, N. Beni, S. Czellar, J. Karancsi, A. Makovec, J. Molnar, Z. Szillasi, M. Bartók, P. Raics, Z. L. Trocsanyi, B. Ujvari, S. Bahinipati, S. Choudhury, P. Mal, K. Mandal, A. Nayak, D. K. Sahoo, N. Sahoo, S. K. Swain, S. Bansal, S. B. Beri, V. Bhatnagar, R. Chawla, U.Bhawandeep, A. K. Kalsi, A. Kaur, M. Kaur, R. Kumar, A. Mehta, M. Mittal, J. B. Singh, G. Walia, Ashok Kumar, A. Bhardwaj, B. C. Choudhary, R. B. Garg, S. Keshri, S. Malhotra, M. Naimuddin, N. Nishu, K. Ranjan, R. Sharma, V. Sharma, R. Bhattacharya, S. Bhattacharya, K. Chatterjee, S. Dey, S. Dutt, S. Dutta, S. Ghosh, N. Majumdar, A. Modak, K. Mondal, S. Mukhopadhyay, S. Nandan, A. Purohit, A. Roy, D. Roy, S. Roy Chowdhury, S. Sarkar, M. Sharan, S. Thakur, P. K. Behera, R. Chudasama, D. Dutta, V. Jha, V. Kumar, A. K. Mohanty, P. K. Netrakanti, L. M. Pant, P. Shukla, A. Topkar, T. Aziz, S. Dugad, G. Kole, B. Mahakud, S. Mitra, G. B. Mohanty, B. Parida, N. Sur, B. Sutar, S. Banerjee, S. Bhowmik, R. K. Dewanjee, S. Ganguly, M. Guchait, Sa. Jain, S. Kumar, M. Maity, G. Majumder, K. Mazumdar, T. Sarkar, N. Wickramage, S. Chauhan, S. Dube, V. Hegde, A. Kapoor, K. Kothekar, A. Rane, S. Sharma, H. Behnamian, S. Chenarani, E. Eskandari Tadavani, S. M. Etesami, A. Fahim, M. Khakzad, M. Mohammadi Najafabadi, M. Naseri, S. Paktinat Mehdiabadi, F. Rezaei Hosseinabadi, B. Safarzadeh, M. Zeinali, M. Felcini, M. Grunewald, M. Abbrescia, C. Calabria, C. Caputo, A. Colaleo, D. Creanza, L. Cristella, N. De Filippis, M. De Palma, L. Fiore, G. Iaselli, G. Maggi, M. Maggi, G. Miniello, S. My, S. Nuzzo, A. Pompili, G. Pugliese, R. Radogna, A. Ranieri, G. Selvaggi, L. Silvestris, R. Venditti, P. Verwilligen, G. Abbiendi, C. Battilana, D. Bonacorsi, S. Braibant-Giacomelli, L. Brigliadori, R. Campanini, P. Capiluppi, A. Castro, F. R. Cavallo, S. S. Chhibra, G. Codispoti, M. Cuffiani, G. M. Dallavalle, F. Fabbri, A. Fanfani, D. Fasanella, P. Giacomelli, C. Grandi, L. Guiducci, S. Marcellini, G. Masetti, A. Montanari, F. L. Navarria, A. Perrotta, A. M. Rossi, T. Rovelli, G. P. Siroli, N. Tosi, S. Albergo, M. Chiorboli, S. Costa, A. Di Mattia, F. Giordano, R. Potenza, A. Tricomi, C. Tuve, G. Barbagli, V. Ciulli, C. Civinini, R. D’Alessandro, E. Focardi, V. Gori, P. Lenzi, M. Meschini, S. Paoletti, G. Sguazzoni, L. Viliani, L. Benussi, S. Bianco, F. Fabbri, D. Piccolo, F. Primavera, V. Calvelli, F. Ferro, M. Lo Vetere, M. R. Monge, E. Robutti, S. Tosi, L. Brianza, M. E. Dinardo, S. Fiorendi, S. Gennai, A. Ghezzi, P. Govoni, S. Malvezzi, R. A. Manzoni, B. Marzocchi, D. Menasce, L. Moroni, M. Paganoni, D. Pedrini, S. Pigazzini, S. Ragazzi, T. Tabarelli de Fatis, S. Buontempo, N. Cavallo, G. De Nardo, S. Di Guida, M. Esposito, F. Fabozzi, A. O. M. Iorio, G. Lanza, L. Lista, S. Meola, P. Paolucci, C. Sciacca, F. Thyssen, P. Azzi, N. Bacchetta, L. Benato, D. Bisello, A. Boletti, R. Carlin, A. Carvalho Antunes De Oliveira, P. Checchia, M. Dall’Osso, P. De Castro Manzano, T. Dorigo, U. Dosselli, F. Gasparini, U. Gasparini, F. Gonella, A. Gozzelino, S. Lacaprara, A. T. Meneguzzo, J. Pazzini, M. Pegoraro, N. Pozzobon, P. Ronchese, M. Sgaravatto, F. Simonetto, E. Torassa, A. Zucchetta, G. Zumerle, A. Braghieri, A. Magnani, P. Montagna, S. P. Ratti, V. Re, C. Riccardi, P. Salvini, I. Vai, P. Vitulo, L. Alunni Solestizi, G. M. Bilei, D. Ciangottini, L. Fanò, P. Lariccia, R. Leonardi, G. Mantovani, M. Menichelli, A. Saha, A. Santocchia, K. Androsov, P. Azzurri, G. Bagliesi, J. Bernardini, T. Boccali, R. Castaldi, M. A. Ciocci, R. Dell’Orso, S. Donato, G. Fedi, A. Giassi, M. T. Grippo, F. Ligabue, T. Lomtadze, L. Martini, A. Messineo, F. Palla, A. Rizzi, A. Savoy-Navarro, P. Spagnolo, R. Tenchini, G. Tonelli, A. Venturi, P. G. Verdini, L. Barone, F. Cavallari, M. Cipriani, G. D’imperio, D. Del Re, M. Diemoz, S. Gelli, C. Jorda, E. Longo, F. Margaroli, P. Meridiani, G. Organtini, R. Paramatti, F. Preiato, S. Rahatlou, C. Rovelli, F. Santanastasio, N. Amapane, R. Arcidiacono, S. Argiro, M. Arneodo, N. Bartosik, R. Bellan, C. Biino, N. Cartiglia, F. Cenna, M. Costa, R. Covarelli, A. Degano, N. Demaria, L. Finco, B. Kiani, C. Mariotti, S. Maselli, E. Migliore, V. Monaco, E. Monteil, M. M. Obertino, L. Pacher, N. Pastrone, M. Pelliccioni, G. L. Pinna Angioni, F. Ravera, A. Romero, M. Ruspa, R. Sacchi, K. Shchelina, V. Sola, A. Solano, A. Staiano, P. Traczyk, S. Belforte, M. Casarsa, F. Cossutti, G. Della Ricca, C. La Licata, A. Schizzi, A. Zanetti, D. H. Kim, G. N. Kim, M. S. Kim, S. Lee, S. W. Lee, Y. D. Oh, S. Sekmen, D. C. Son, Y. C. Yang, A. Lee, J. A. Brochero Cifuentes, T. J. Kim, S. Cho, S. Choi, Y. Go, D. Gyun, S. Ha, B. Hong, Y. Jo, Y. Kim, B. Lee, K. Lee, K. S. Lee, S. Lee, J. Lim, S. K. Park, Y. Roh, J. Almond, J. Kim, S. B. Oh, S. h. Seo, U. K. Yang, H. D. Yoo, G. B. Yu, M. Choi, H. Kim, H. Kim, J. H. Kim, J. S. H. Lee, I. C. Park, G. Ryu, M. S. Ryu, Y. Choi, J. Goh, C. Hwang, J. Lee, I. Yu, V. Dudenas, A. Juodagalvis, J. Vaitkus, I. Ahmed, Z. A. Ibrahim, J. R. Komaragiri, M. A. B. Md Ali, F. Mohamad Idris, W. A. T. Wan Abdullah, M. N. Yusli, Z. Zolkapli, H. Castilla-Valdez, E. De La Cruz-Burelo, I. Heredia-De La Cruz, A. Hernandez-Almada, R. Lopez-Fernandez, R. Magaña Villalba, J. Mejia Guisao, A. Sanchez-Hernandez, S. Carrillo Moreno, C. Oropeza Barrera, F. Vazquez Valencia, S. Carpinteyro, I. Pedraza, H. A. Salazar Ibarguen, C. Uribe Estrada, A. Morelos Pineda, D. Krofcheck, P. H. Butler, A. Ahmad, M. Ahmad, Q. Hassan, H. R. Hoorani, W. A. Khan, M. A. Shah, M. Shoaib, M. Waqas, H. Bialkowska, M. Bluj, B. Boimska, T. Frueboes, M. Górski, M. Kazana, K. Nawrocki, K. Romanowska-Rybinska, M. Szleper, P. Zalewski, K. Bunkowski, A. Byszuk, K. Doroba, A. Kalinowski, M. Konecki, J. Krolikowski, M. Misiura, M. Olszewski, M. Walczak, P. Bargassa, C. Beirão Da Cruz E Silva, A. Di Francesco, P. Faccioli, P. G. Ferreira Parracho, M. Gallinaro, J. Hollar, N. Leonardo, L. Lloret Iglesias, M. V. Nemallapudi, J. Rodrigues Antunes, J. Seixas, O. Toldaiev, D. Vadruccio, J. Varela, P. Vischia, A. Golunov, I. Golutvin, N. Gorbounov, V. Karjavin, V. Korenkov, A. Lanev, A. Malakhov, V. Matveev, V. V. Mitsyn, P. Moisenz, V. Palichik, V. Perelygin, S. Shmatov, S. Shulha, N. Skatchkov, V. Smirnov, E. Tikhonenko, B. S. Yuldashev, A. Zarubin, L. Chtchipounov, V. Golovtsov, Y. Ivanov, V. Kim, E. Kuznetsova, V. Murzin, V. Oreshkin, V. Sulimov, A. Vorobyev, Yu. Andreev, A. Dermenev, S. Gninenko, N. Golubev, A. Karneyeu, M. Kirsanov, N. Krasnikov, A. Pashenkov, D. Tlisov, A. Toropin, V. Epshteyn, V. Gavrilov, N. Lychkovskaya, V. Popov, I. Pozdnyakov, G. Safronov, A. Spiridonov, M. Toms, E. Vlasov, A. Zhokin, A. Bylinkin, M. Chadeeva, E. Popova, E. Tarkovskii, V. Andreev, M. Azarkin, I. Dremin, M. Kirakosyan, A. Leonidov, S. V. Rusakov, A. Terkulov, A. Baskakov, A. Belyaev, E. Boos, V. Bunichev, M. Dubinin, L. Dudko, A. Gribushin, V. Klyukhin, O. Kodolova, I. Lokhtin, I. Miagkov, S. Obraztsov, M. Perfilov, V. Savrin, A. Snigirev, V. Blinov, Y. Skovpen, I. Azhgirey, I. Bayshev, S. Bitioukov, D. Elumakhov, V. Kachanov, A. Kalinin, D. Konstantinov, V. Krychkine, V. Petrov, R. Ryutin, A. Sobol, S. Troshin, N. Tyurin, A. Uzunian, A. Volkov, P. Adzic, P. Cirkovic, D. Devetak, M. Dordevic, J. Milosevic, V. Rekovic, J. Alcaraz Maestre, M. Barrio Luna, E. Calvo, M. Cerrada, M. Chamizo Llatas, N. Colino, B. De La Cruz, A. Delgado Peris, A. Escalante Del Valle, C. Fernandez Bedoya, J. P. Fernández Ramos, J. Flix, M. C. Fouz, P. Garcia-Abia, O. Gonzalez Lopez, S. Goy Lopez, J. M. Hernandez, M. I. Josa, E. Navarro De Martino, A. Pérez-Calero Yzquierdo, J. Puerta Pelayo, A. Quintario Olmeda, I. Redondo, L. Romero, M. S. Soares, J. F. de Trocóniz, M. Missiroli, D. Moran, J. Cuevas, J. Fernandez Menendez, I. Gonzalez Caballero, J. R. González Fernández, E. Palencia Cortezon, S. Sanchez Cruz, I. Suárez Andrés, J. M. Vizan Garcia, I. J. Cabrillo, A. Calderon, J. R. Castiñeiras De Saa, E. Curras, M. Fernandez, J. Garcia-Ferrero, G. Gomez, A. Lopez Virto, J. Marco, C. Martinez Rivero, F. Matorras, J. Piedra Gomez, T. Rodrigo, A. Ruiz-Jimeno, L. Scodellaro, N. Trevisani, I. Vila, R. Vilar Cortabitarte, D. Abbaneo, E. Auffray, G. Auzinger, M. Bachtis, P. Baillon, A. H. Ball, D. Barney, P. Bloch, A. Bocci, A. Bonato, C. Botta, T. Camporesi, R. Castello, M. Cepeda, G. Cerminara, M. D’Alfonso, D. d’Enterria, A. Dabrowski, V. Daponte, A. David, M. De Gruttola, F. De Guio, A. De Roeck, E. Di Marco, M. Dobson, B. Dorney, T. du Pree, D. Duggan, M. Dünser, N. Dupont, A. Elliott-Peisert, S. Fartoukh, G. Franzoni, J. Fulcher, W. Funk, D. Gigi, K. Gill, M. Girone, F. Glege, D. Gulhan, S. Gundacker, M. Guthoff, J. Hammer, P. Harris, J. Hegeman, V. Innocente, P. Janot, H. Kirschenmann, V. Knünz, A. Kornmayer, M. J. Kortelainen, K. Kousouris, M. Krammer, P. Lecoq, C. Lourenço, M. T. Lucchini, L. Malgeri, M. Mannelli, A. Martelli, F. Meijers, S. Mersi, E. Meschi, F. Moortgat, S. Morovic, M. Mulders, H. Neugebauer, S. Orfanelli, L. Orsini, L. Pape, E. Perez, M. Peruzzi, A. Petrilli, G. Petrucciani, A. Pfeiffer, M. Pierini, A. Racz, T. Reis, G. Rolandi, M. Rovere, M. Ruan, H. Sakulin, J. B. Sauvan, C. Schäfer, C. Schwick, M. Seidel, A. Sharma, P. Silva, M. Simon, P. Sphicas, J. Steggemann, M. Stoye, Y. Takahashi, M. Tosi, D. Treille, A. Triossi, A. Tsirou, V. Veckalns, G. I. Veres, N. Wardle, A. Zagozdzinska, W. D. Zeuner, W. Bertl, K. Deiters, W. Erdmann, R. Horisberger, Q. Ingram, H. C. Kaestli, D. Kotlinski, U. Langenegger, T. Rohe, F. Bachmair, L. Bäni, L. Bianchini, B. Casal, G. Dissertori, M. Dittmar, M. Donegà, P. Eller, C. Grab, C. Heidegger, D. Hits, J. Hoss, G. Kasieczka, P. Lecomte, W. Lustermann, B. Mangano, M. Marionneau, P. Martinez Ruiz del Arbol, M. Masciovecchio, M. T. Meinhard, D. Meister, F. Micheli, P. Musella, F. Nessi-Tedaldi, F. Pandolfi, J. Pata, F. Pauss, G. Perrin, L. Perrozzi, M. Quittnat, M. Rossini, M. Schönenberger, A. Starodumov, V. R. Tavolaro, K. Theofilatos, R. Wallny, T. K. Aarrestad, C. Amsler, L. Caminada, M. F. Canelli, A. De Cosa, C. Galloni, A. Hinzmann, T. Hreus, B. Kilminster, C. Lange, J. Ngadiuba, D. Pinna, G. Rauco, P. Robmann, D. Salerno, Y. Yang, V. Candelise, T. H. Doan, Sh. Jain, R. Khurana, M. Konyushikhin, C. M. Kuo, W. Lin, Y. J. Lu, A. Pozdnyakov, S. S. Yu, Arun Kumar, P. Chang, Y. H. Chang, Y. W. Chang, Y. Chao, K. F. Chen, P. H. Chen, C. Dietz, F. Fiori, W. -S. Hou, Y. Hsiung, Y. F. Liu, R. -S. Lu, M. Miñano Moya, E. Paganis, A. Psallidas, J. f. Tsai, Y. M. Tzeng, B. Asavapibhop, G. Singh, N. Srimanobhas, N. Suwonjandee, A. Adiguzel, M. N. Bakirci, S. Damarseckin, Z. S. Demiroglu, C. Dozen, E. Eskut, S. Girgis, G. Gokbulut, Y. Guler, E. Gurpinar, I. Hos, E. E. Kangal, O. Kara, U. Kiminsu, M. Oglakci, G. Onengut, K. Ozdemir, S. Ozturk, A. Polatoz, D. Sunar Cerci, S. Turkcapar, I. S. Zorbakir, C. Zorbilmez, B. Bilin, S. Bilmis, B. Isildak, G. Karapinar, M. Yalvac, M. Zeyrek, E. Gülmez, M. Kaya, O. Kaya, E. A. Yetkin, T. Yetkin, A. Cakir, K. Cankocak, S. Sen, B. Grynyov, L. Levchuk, P. Sorokin, R. Aggleton, F. Ball, L. Beck, J. J. Brooke, D. Burns, E. Clement, D. Cussans, H. Flacher, J. Goldstein, M. Grimes, G. P. Heath, H. F. Heath, J. Jacob, L. Kreczko, C. Lucas, D. M. Newbold, S. Paramesvaran, A. Poll, T. Sakuma, S. Seif El Nasr-storey, D. Smith, V. J. Smith, K. W. Bell, A. Belyaev, C. Brew, R. M. Brown, L. Calligaris, D. Cieri, D. J. A. Cockerill, J. A. Coughlan, K. Harder, S. Harper, E. Olaiya, D. Petyt, C. H. Shepherd-Themistocleous, A. Thea, I. R. Tomalin, T. Williams, M. Baber, R. Bainbridge, O. Buchmuller, A. Bundock, D. Burton, S. Casasso, M. Citron, D. Colling, L. Corpe, P. Dauncey, G. Davies, A. De Wit, M. Della Negra, R. Di Maria, P. Dunne, A. Elwood, D. Futyan, Y. Haddad, G. Hall, G. Iles, T. James, R. Lane, C. Laner, R. Lucas, L. Lyons, A. -M. Magnan, S. Malik, L. Mastrolorenzo, J. Nash, A. Nikitenko, J. Pela, B. Penning, M. Pesaresi, D. M. Raymond, A. Richards, A. Rose, C. Seez, S. Summers, A. Tapper, K. Uchida, M. Vazquez Acosta, T. Virdee, J. Wright, S. C. Zenz, J. E. Cole, P. R. Hobson, A. Khan, P. Kyberd, D. Leslie, I. D. Reid, P. Symonds, L. Teodorescu, M. Turner, A. Borzou, K. Call, J. Dittmann, K. Hatakeyama, H. Liu, N. Pastika, O. Charaf, S. I. Cooper, C. Henderson, P. Rumerio, D. Arcaro, A. Avetisyan, T. Bose, D. Gastler, D. Rankin, C. Richardson, J. Rohlf, L. Sulak, D. Zou, G. Benelli, E. Berry, D. Cutts, A. Garabedian, J. Hakala, U. Heintz, J. M. Hogan, O. Jesus, E. Laird, G. Landsberg, Z. Mao, M. Narain, S. Piperov, S. Sagir, E. Spencer, R. Syarif, R. Breedon, G. Breto, D. Burns, M. Calderon De La Barca Sanchez, S. Chauhan, M. Chertok, J. Conway, R. Conway, P. T. Cox, R. Erbacher, C. Flores, G. Funk, M. Gardner, W. Ko, R. Lander, C. Mclean, M. Mulhearn, D. Pellett, J. Pilot, F. Ricci-Tam, S. Shalhout, J. Smith, M. Squires, D. Stolp, M. Tripathi, S. Wilbur, R. Yohay, R. Cousins, P. Everaerts, A. Florent, J. Hauser, M. Ignatenko, D. Saltzberg, E. Takasugi, V. Valuev, M. Weber, K. Burt, R. Clare, J. Ellison, J. W. Gary, G. Hanson, J. Heilman, P. Jandir, E. Kennedy, F. Lacroix, O. R. Long, M. Malberti, M. Olmedo Negrete, M. I. Paneva, A. Shrinivas, H. Wei, S. Wimpenny, B. R. Yates, J. G. Branson, G. B. Cerati, S. Cittolin, M. Derdzinski, R. Gerosa, A. Holzner, D. Klein, V. Krutelyov, J. Letts, I. Macneill, D. Olivito, S. Padhi, M. Pieri, M. Sani, V. Sharma, S. Simon, M. Tadel, A. Vartak, S. Wasserbaech, C. Welke, J. Wood, F. Würthwein, A. Yagil, G. Zevi Della Porta, R. Bhandari, J. Bradmiller-Feld, C. Campagnari, A. Dishaw, V. Dutta, K. Flowers, M. Franco Sevilla, P. Geffert, C. George, F. Golf, L. Gouskos, J. Gran, R. Heller, J. Incandela, N. Mccoll, S. D. Mullin, A. Ovcharova, J. Richman, D. Stuart, I. Suarez, C. West, J. Yoo, D. Anderson, A. Apresyan, J. Bendavid, A. Bornheim, J. Bunn, Y. Chen, J. Duarte, J. M. Lawhorn, A. Mott, H. B. Newman, C. Pena, M. Spiropulu, J. R. Vlimant, S. Xie, R. Y. Zhu, M. B. Andrews, V. Azzolini, B. Carlson, T. Ferguson, M. Paulini, J. Russ, M. Sun, H. Vogel, I. Vorobiev, J. P. Cumalat, W. T. Ford, F. Jensen, A. Johnson, M. Krohn, T. Mulholland, K. Stenson, S. R. Wagner, J. Alexander, J. Chaves, J. Chu, S. Dittmer, K. Mcdermott, N. Mirman, G. Nicolas Kaufman, J. R. Patterson, A. Rinkevicius, A. Ryd, L. Skinnari, L. Soffi, S. M. Tan, Z. Tao, J. Thom, J. Tucker, P. Wittich, M. Zientek, D. Winn, S. Abdullin, M. Albrow, G. Apollinari, S. Banerjee, L. A. T. Bauerdick, A. Beretvas, J. Berryhill, P. C. Bhat, G. Bolla, K. Burkett, J. N. Butler, H. W. K. Cheung, F. Chlebana, S. Cihangir, M. Cremonesi, V. D. Elvira, I. Fisk, J. Freeman, E. Gottschalk, L. Gray, D. Green, S. Grünendahl, O. Gutsche, D. Hare, R. M. Harris, S. Hasegawa, J. Hirschauer, Z. Hu, B. Jayatilaka, S. Jindariani, M. Johnson, U. Joshi, B. Klima, B. Kreis, S. Lammel, J. Linacre, D. Lincoln, R. Lipton, T. Liu, R. Lopes De Sá, J. Lykken, K. Maeshima, N. Magini, J. M. Marraffino, S. Maruyama, D. Mason, P. McBride, P. Merkel, S. Mrenna, S. Nahn, C. Newman-Holmes, V. O’Dell, K. Pedro, O. Prokofyev, G. Rakness, L. Ristori, E. Sexton-Kennedy, A. Soha, W. J. Spalding, L. Spiegel, S. Stoynev, N. Strobbe, L. Taylor, S. Tkaczyk, N. V. Tran, L. Uplegger, E. W. Vaandering, C. Vernieri, M. Verzocchi, R. Vidal, M. Wang, H. A. Weber, A. Whitbeck, D. Acosta, P. Avery, P. Bortignon, D. Bourilkov, A. Brinkerhoff, A. Carnes, M. Carver, D. Curry, S. Das, R. D. Field, I. K. Furic, J. Konigsberg, A. Korytov, P. Ma, K. Matchev, H. Mei, P. Milenovic, G. Mitselmakher, D. Rank, L. Shchutska, D. Sperka, L. Thomas, J. Wang, S. Wang, J. Yelton, S. Linn, P. Markowitz, G. Martinez, J. L. Rodriguez, A. Ackert, J. R. Adams, T. Adams, A. Askew, S. Bein, B. Diamond, S. Hagopian, V. Hagopian, K. F. Johnson, A. Khatiwada, H. Prosper, A. Santra, M. Weinberg, M. M. Baarmand, V. Bhopatkar, S. Colafranceschi, M. Hohlmann, D. Noonan, T. Roy, F. Yumiceva, M. R. Adams, L. Apanasevich, D. Berry, R. R. Betts, I. Bucinskaite, R. Cavanaugh, O. Evdokimov, L. Gauthier, C. E. Gerber, D. J. Hofman, P. Kurt, C. O’Brien, I. D. Sandoval Gonzalez, P. Turner, N. Varelas, H. Wang, Z. Wu, M. Zakaria, J. Zhang, B. Bilki, W. Clarida, K. Dilsiz, S. Durgut, R. P. Gandrajula, M. Haytmyradov, V. Khristenko, J. -P. Merlo, H. Mermerkaya, A. Mestvirishvili, A. Moeller, J. Nachtman, H. Ogul, Y. Onel, F. Ozok, A. Penzo, C. Snyder, E. Tiras, J. Wetzel, K. Yi, I. Anderson, B. Blumenfeld, A. Cocoros, N. Eminizer, D. Fehling, L. Feng, A. V. Gritsan, P. Maksimovic, M. Osherson, J. Roskes, U. Sarica, M. Swartz, M. Xiao, Y. Xin, C. You, A. Al-bataineh, P. Baringer, A. Bean, J. Bowen, C. Bruner, J. Castle, R. P. Kenny III, A. Kropivnitskaya, D. Majumder, W. Mcbrayer, M. Murray, S. Sanders, R. Stringer, J. D. Tapia Takaki, Q. Wang, A. Ivanov, K. Kaadze, S. Khalil, M. Makouski, Y. Maravin, A. Mohammadi, L. K. Saini, N. Skhirtladze, S. Toda, D. Lange, F. Rebassoo, D. Wright, C. Anelli, A. Baden, O. Baron, A. Belloni, B. Calvert, S. C. Eno, C. Ferraioli, J. A. Gomez, N. J. Hadley, S. Jabeen, R. G. Kellogg, T. Kolberg, J. Kunkle, Y. Lu, A. C. Mignerey, Y. H. Shin, A. Skuja, M. B. Tonjes, S. C. Tonwar, D. Abercrombie, B. Allen, A. Apyan, R. Barbieri, A. Baty, R. Bi, K. Bierwagen, S. Brandt, W. Busza, I. A. Cali, Z. Demiragli, L. Di Matteo, G. Gomez Ceballos, M. Goncharov, D. Hsu, Y. Iiyama, G. M. Innocenti, M. Klute, D. Kovalskyi, K. Krajczar, Y. S. Lai, Y. -J. Lee, A. Levin, P. D. Luckey, A. C. Marini, C. Mcginn, C. Mironov, S. Narayanan, X. Niu, C. Paus, C. Roland, G. Roland, J. Salfeld-Nebgen, G. S. F. Stephans, K. Sumorok, K. Tatar, M. Varma, D. Velicanu, J. Veverka, J. Wang, T. W. Wang, B. Wyslouch, M. Yang, V. Zhukova, A. C. Benvenuti, R. M. Chatterjee, A. Evans, A. Finkel, A. Gude, P. Hansen, S. Kalafut, S. C. Kao, Y. Kubota, Z. Lesko, J. Mans, S. Nourbakhsh, N. Ruckstuhl, R. Rusack, N. Tambe, J. Turkewitz, J. G. Acosta, S. Oliveros, E. Avdeeva, R. Bartek, K. Bloom, S. Bose, D. R. Claes, A. Dominguez, C. Fangmeier, R. Gonzalez Suarez, R. Kamalieddin, D. Knowlton, I. Kravchenko, A. Malta Rodrigues, F. Meier, J. Monroy, J. E. Siado, G. R. Snow, B. Stieger, M. Alyari, J. Dolen, J. George, A. Godshalk, C. Harrington, I. Iashvili, J. Kaisen, A. Kharchilava, A. Kumar, A. Parker, S. Rappoccio, B. Roozbahani, G. Alverson, E. Barberis, D. Baumgartel, A. Hortiangtham, A. Massironi, D. M. Morse, D. Nash, T. Orimoto, R. Teixeira De Lima, D. Trocino, R. -J. Wang, D. Wood, S. Bhattacharya, K. A. Hahn, A. Kubik, A. Kumar, J. F. Low, N. Mucia, N. Odell, B. Pollack, M. H. Schmitt, K. Sung, M. Trovato, M. Velasco, N. Dev, M. Hildreth, K. Hurtado Anampa, C. Jessop, D. J. Karmgard, N. Kellams, K. Lannon, N. Marinelli, F. Meng, C. Mueller, Y. Musienko, M. Planer, A. Reinsvold, R. Ruchti, G. Smith, S. Taroni, N. Valls, M. Wayne, M. Wolf, A. Woodard, J. Alimena, L. Antonelli, J. Brinson, B. Bylsma, L. S. Durkin, S. Flowers, B. Francis, A. Hart, C. Hill, R. Hughes, W. Ji, B. Liu, W. Luo, D. Puigh, B. L. Winer, H. W. Wulsin, S. Cooperstein, O. Driga, P. Elmer, J. Hardenbrook, P. Hebda, J. Luo, D. Marlow, T. Medvedeva, K. Mei, M. Mooney, J. Olsen, C. Palmer, P. Piroué, D. Stickland, C. Tully, A. Zuranski, S. Malik, A. Barker, V. E. Barnes, S. Folgueras, L. Gutay, M. K. Jha, M. Jones, A. W. Jung, K. Jung, D. H. Miller, N. Neumeister, B. C. Radburn-Smith, X. Shi, J. Sun, A. Svyatkovskiy, F. Wang, W. Xie, L. Xu, N. Parashar, J. Stupak, A. Adair, B. Akgun, Z. Chen, K. M. Ecklund, F. J. M. Geurts, M. Guilbaud, W. Li, B. Michlin, M. Northup, B. P. Padley, R. Redjimi, J. Roberts, J. Rorie, Z. Tu, J. Zabel, B. Betchart, A. Bodek, P. de Barbaro, R. Demina, Y. t. Duh, T. Ferbel, M. Galanti, A. Garcia-Bellido, J. Han, O. Hindrichs, A. Khukhunaishvili, K. H. Lo, P. Tan, M. Verzetti, J. P. Chou, E. Contreras-Campana, Y. Gershtein, T. A. Gómez Espinosa, E. Halkiadakis, M. Heindl, D. Hidas, E. Hughes, S. Kaplan, R. Kunnawalkam Elayavalli, S. Kyriacou, A. Lath, K. Nash, H. Saka, S. Salur, S. Schnetzer, D. Sheffield, S. Somalwar, R. Stone, S. Thomas, P. Thomassen, M. Walker, M. Foerster, J. Heideman, G. Riley, K. Rose, S. Spanier, K. Thapa, O. Bouhali, A. Celik, M. Dalchenko, M. De Mattia, A. Delgado, S. Dildick, R. Eusebi, J. Gilmore, T. Huang, E. Juska, T. Kamon, R. Mueller, Y. Pakhotin, R. Patel, A. Perloff, L. Perniè, D. Rathjens, A. Rose, A. Safonov, A. Tatarinov, K. A. Ulmer, N. Akchurin, C. Cowden, J. Damgov, C. Dragoiu, P. R. Dudero, J. Faulkner, S. Kunori, K. Lamichhane, S. W. Lee, T. Libeiro, S. Undleeb, I. Volobouev, Z. Wang, A. G. Delannoy, S. Greene, A. Gurrola, R. Janjam, W. Johns, C. Maguire, A. Melo, H. Ni, P. Sheldon, S. Tuo, J. Velkovska, Q. Xu, M. W. Arenton, P. Barria, B. Cox, J. Goodell, R. Hirosky, A. Ledovskoy, H. Li, C. Neu, T. Sinthuprasith, X. Sun, Y. Wang, E. Wolfe, F. Xia, C. Clarke, R. Harr, P. E. Karchin, P. Lamichhane, J. Sturdy, D. A. Belknap, S. Dasu, L. Dodd, S. Duric, B. Gomber, M. Grothe, M. Herndon, A. Hervé, P. Klabbers, A. Lanaro, A. Levine, K. Long, R. Loveless, I. Ojalvo, T. Perry, G. A. Pierro, G. Polese, T. Ruggles, A. Savin, A. Sharma, N. Smith, W. H. Smith, D. Taylor, N. Woods, CMS Collaboration

**Affiliations:** 1 Yerevan Physics Institute, Yerevan, Armenia; 2 Institut für Hochenergiephysik der OeAW, Wien, Austria; 3 National Centre for Particle and High Energy Physics, Minsk, Belarus; 4 Universiteit Antwerpen, Antwerpen, Belgium; 5 Vrije Universiteit Brussel, Brussel, Belgium; 6 Université Libre de Bruxelles, Bruxelles, Belgium; 7 Ghent University, Ghent, Belgium; 8 Université Catholique de Louvain, Louvain-la-Neuve, Belgium; 9 Université de Mons, Mons, Belgium; 10 Centro Brasileiro de Pesquisas Fisicas, Rio de Janeiro, Brazil; 11 Universidade do Estado do Rio de Janeiro, Rio de Janeiro, Brazil; 12 Universidade Estadual Paulista , Universidade Federal do ABC, São Paulo, Brazil; 13 Institute for Nuclear Research and Nuclear Energy, Sofia, Bulgaria; 14 University of Sofia, Sofia, Bulgaria; 15 Beihang University, Beijing, China; 16 Institute of High Energy Physics, Beijing, China; 17 State Key Laboratory of Nuclear Physics and Technology, Peking University, Beijing, China; 18 Universidad de Los Andes, Bogota, Colombia; 19 Faculty of Electrical Engineering, Mechanical Engineering and Naval Architecture, University of Split, Split, Croatia; 20 Faculty of Science, University of Split, Split, Croatia; 21 Institute Rudjer Boskovic, Zagreb, Croatia; 22 University of Cyprus, Nicosia, Cyprus; 23 Charles University, Prague, Czech Republic; 24 Universidad San Francisco de Quito, Quito, Ecuador; 25 Academy of Scientific Research and Technology of the Arab Republic of Egypt, Egyptian Network of High Energy Physics, Cairo, Egypt; 26 National Institute of Chemical Physics and Biophysics, Tallinn, Estonia; 27 Department of Physics, University of Helsinki, Helsinki, Finland; 28 Helsinki Institute of Physics, Helsinki, Finland; 29 Lappeenranta University of Technology, Lappeenranta, Finland; 30 IRFU, CEA, Université Paris-Saclay, Gif-sur-Yvette, France; 31 Laboratoire Leprince-Ringuet, Ecole Polytechnique, IN2P3-CNRS, Palaiseau, France; 32 Institut Pluridisciplinaire Hubert Curien, Université de Strasbourg, Université de Haute Alsace Mulhouse, CNRS/IN2P3 Strasbourg, France; 33 Centre de Calcul de l’Institut National de Physique Nucleaire et de Physique des Particules, CNRS/IN2P3 Villeurbanne, France; 34 Institut de Physique Nucléaire de Lyon, Université de Lyon, Université Claude Bernard Lyon 1, CNRS-IN2P3, Villeurbanne, France; 35 Georgian Technical University, Tbilisi, Georgia; 36 Tbilisi State University, Tbilisi, Georgia; 37 I. Physikalisches Institut, RWTH Aachen University, Aachen, Germany; 38 III. Physikalisches Institut A, RWTH Aachen University, Aachen, Germany; 39 III. Physikalisches Institut B, RWTH Aachen University, Aachen, Germany; 40 Deutsches Elektronen-Synchrotron, Hamburg, Germany; 41 University of Hamburg, Hamburg, Germany; 42 Institut für Experimentelle Kernphysik, Karlsruhe, Germany; 43 Institute of Nuclear and Particle Physics (INPP), NCSR Demokritos, Aghia Paraskevi, Greece; 44 National and Kapodistrian University of Athens, Athens, Greece; 45 University of Ioánnina, Ioannina, Greece; 46 MTA-ELTE Lendület CMS Particle and Nuclear Physics Group, Eötvös Loránd University, Budapest, Hungary; 47 Wigner Research Centre for Physics, Budapest, Hungary; 48 Institute of Nuclear Research ATOMKI, Debrecen, Hungary; 49 University of Debrecen, Debrecen, Hungary; 50 National Institute of Science Education and Research, Bhubaneswar, India; 51 Panjab University, Chandigarh, India; 52 University of Delhi, Delhi, India; 53 Saha Institute of Nuclear Physics, Kolkata, India; 54 Indian Institute of Technology Madras, Madras, India; 55 Bhabha Atomic Research Centre, Mumbai, India; 56 Tata Institute of Fundamental Research-A, Mumbai, India; 57 Tata Institute of Fundamental Research-B, Mumbai, India; 58 Indian Institute of Science Education and Research (IISER), Pune, India; 59 Institute for Research in Fundamental Sciences (IPM), Tehran, Iran; 60 University College Dublin, Dublin, Ireland; 61 INFN Sezione di Bari , Università di Bari , Politecnico di Bari, Bari, Italy; 62 INFN Sezione di Bologna , Università di Bologna, Bologna, Italy; 63 INFN Sezione di Catania , Università di Catania, Catania, Italy; 64 INFN Sezione di Firenze , Università di Firenze, Firenze, Italy; 65 INFN Laboratori Nazionali di Frascati, Frascati, Italy; 66 INFN Sezione di Genova , Università di Genova, Genoa, Italy; 67 INFN Sezione di Milano-Bicocca , Università di Milano-Bicocca, Milan, Italy; 68 INFN Sezione di Napoli , Università di Napoli ’Federico II’ Napoli, Italy, Università della Basilicata , Potenza, Italy, Università G. Marconi, Rome, Italy; 69 INFN Sezione di Padova , Università di Padova , Padova, Italy, Università di Trento, Trento, Italy; 70 INFN Sezione di Pavia , Università di Pavia, Pavia, Italy; 71 INFN Sezione di Perugia , Università di Perugia, Perugia, Italy; 72 INFN Sezione di Pisa , Università di Pisa , Scuola Normale Superiore di Pisa, Pisa, Italy; 73 INFN Sezione di Roma , Università di Roma, Rome, Italy; 74 INFN Sezione di Torino , Università di Torino , Turin, Italy, Università del Piemonte Orientale, Novara, Italy; 75 INFN Sezione di Trieste , Università di Trieste, Trieste, Italy; 76 Kyungpook National University, Taegu, Korea; 77 Chonbuk National University, Chonju, Korea; 78 Hanyang University, Seoul, Korea; 79 Korea University, Seoul, Korea; 80 Seoul National University, Seoul, Korea; 81 University of Seoul, Seoul, Korea; 82 Sungkyunkwan University, Suwon, Korea; 83 Vilnius University, Vilnius, Lithuania; 84 National Centre for Particle Physics, Universiti Malaya, Kuala Lumpur, Malaysia; 85 Centro de Investigacion y de Estudios Avanzados del IPN, Mexico City, Mexico; 86 Universidad Iberoamericana, Mexico City, Mexico; 87 Benemerita Universidad Autonoma de Puebla, Puebla, Mexico; 88 Universidad Autónoma de San Luis Potosí, San Luis Potosí, Mexico; 89 University of Auckland, Auckland, New Zealand; 90 University of Canterbury, Christchurch, New Zealand; 91 National Centre for Physics, Quaid-I-Azam University, Islamabad, Pakistan; 92 National Centre for Nuclear Research, Swierk, Poland; 93 Institute of Experimental Physics, Faculty of Physics, University of Warsaw, Warsaw, Poland; 94 Laboratório de Instrumentação e Física Experimental de Partículas, Lisbon, Portugal; 95 Joint Institute for Nuclear Research, Dubna, Russia; 96 Petersburg Nuclear Physics Institute, Gatchina (St. Petersburg), Russia; 97 Institute for Nuclear Research, Moscow, Russia; 98 Institute for Theoretical and Experimental Physics, Moscow, Russia; 99 Moscow Institute of Physics and Technology, Dolgoprudny, Russia; 100 National Research Nuclear University ’Moscow Engineering Physics Institute’ (MEPhI), Moscow, Russia; 101 P.N. Lebedev Physical Institute, Moscow, Russia; 102 Skobeltsyn Institute of Nuclear Physics, Lomonosov Moscow State University, Moscow, Russia; 103 Novosibirsk State University (NSU), Novosibirsk, Russia; 104 State Research Center of Russian Federation, Institute for High Energy Physics, Protvino, Russia; 105 Faculty of Physics and Vinca Institute of Nuclear Sciences, University of Belgrade, Belgrade, Serbia; 106 Centro de Investigaciones Energéticas Medioambientales y Tecnológicas (CIEMAT), Madrid, Spain; 107 Universidad Autónoma de Madrid, Madrid, Spain; 108 Universidad de Oviedo, Oviedo, Spain; 109 Instituto de Física de Cantabria (IFCA), CSIC-Universidad de Cantabria, Santander, Spain; 110 CERN, European Organization for Nuclear Research, Geneva, Switzerland; 111 Paul Scherrer Institut, Villigen, Switzerland; 112 Institute for Particle Physics, ETH Zurich, Zurich Switzerland; 113 Universität Zürich, Zurich, Switzerland; 114 National Central University, Chung-Li, Taiwan; 115 National Taiwan University (NTU), Taipei, Taiwan; 116 Department of Physics, Faculty of Science, Chulalongkorn University, Bangkok, Thailand; 117 Cukurova University, Adana, Turkey; 118 Physics Department, Middle East Technical University, Ankara, Turkey; 119 Bogazici University, Istanbul, Turkey; 120 Istanbul Technical University, Istanbul, Turkey; 121 Institute for Scintillation Materials of National Academy of Science of Ukraine, Kharkov, Ukraine; 122 National Scientific Center, Kharkov Institute of Physics and Technology, Kharkov, Ukraine; 123 University of Bristol, Bristol, United Kingdom; 124 Rutherford Appleton Laboratory, Didcot, UK; 125 Imperial College, London, UK; 126 Brunel University, Uxbridge, UK; 127 Baylor University, Waco, USA; 128 The University of Alabama, Tuscaloosa, USA; 129 Boston University, Boston, USA; 130 Brown University, Providence, USA; 131 University of California, Davis, Davis USA; 132 University of California, Los Angeles, USA; 133 University of California, Riverside, Riverside USA; 134 University of California, San Diego, La Jolla USA; 135 University of California, Santa Barbara - Department of Physics, Santa Barbara, USA; 136 California Institute of Technology, Pasadena, USA; 137 Carnegie Mellon University, Pittsburgh, USA; 138 University of Colorado Boulder, Boulder, USA; 139 Cornell University, Ithaca, USA; 140 Fairfield University, Fairfield, USA; 141 Fermi National Accelerator Laboratory, Batavia, USA; 142 University of Florida, Gainesville, USA; 143 Florida International University, Miami, USA; 144 Florida State University, Tallahassee, USA; 145 Florida Institute of Technology, Melbourne, USA; 146 University of Illinois at Chicago (UIC), Chicago, USA; 147 The University of Iowa, Iowa City, USA; 148 Johns Hopkins University, Baltimore, USA; 149 The University of Kansas, Lawrence, USA; 150 Kansas State University, Manhattan, USA; 151 Lawrence Livermore National Laboratory, Livermore, USA; 152 University of Maryland, College Park, USA; 153 Massachusetts Institute of Technology, Cambridge, USA; 154 University of Minnesota, Minneapolis, USA; 155 University of Mississippi, Oxford, USA; 156 University of Nebraska-Lincoln, Lincoln, USA; 157 State University of New York at Buffalo, Buffalo, USA; 158 Northeastern University, Boston, USA; 159 Northwestern University, Evanston, USA; 160 University of Notre Dame, Notre Dame, USA; 161 The Ohio State University, Columbus, USA; 162 Princeton University, Princeton, USA; 163 University of Puerto Rico, Mayaguez, USA; 164 Purdue University, West Lafayette, USA; 165 Purdue University Calumet, Hammond, USA; 166 Rice University, Houston, USA; 167 University of Rochester, Rochester, USA; 168 Rutgers, The State University of New Jersey, Piscataway, USA; 169 University of Tennessee, Knoxville, USA; 170 Texas A&M University, College Station, USA; 171 Texas Tech University, Lubbock, USA; 172 Vanderbilt University, Nashville, USA; 173 University of Virginia, Charlottesville, USA; 174 Wayne State University, Detroit, USA; 175 University of Wisconsin-Madison, Madison, WI USA; 176 CERN, Geneva, Switzerland

## Abstract

The production cross section of a W boson in association with two b jets is measured using a sample of proton–proton collisions at $\sqrt{s} = 8{\,\mathrm{{TeV}}}$ collected by the CMS experiment at the CERN LHC. The data sample corresponds to an integrated luminosity of 19.8$\,\text {fb}^\text {-1}$. The W bosons are reconstructed via their leptonic decays, $\mathrm {W}\rightarrow \ell \nu$, where $\ell =\mu$ or $\mathrm {e}$. The fiducial region studied contains exactly one lepton with transverse momentum $p_{\mathrm {T}} ^{\ell }>30\,\mathrm{{GeV}}$ and pseudorapidity $|\eta ^{\ell } |<2.1$, with exactly two b jets with $p_{\mathrm {T}} >25\,\mathrm{{GeV}}$ and $|\eta |<2.4$ and no other jets with $p_{\mathrm {T}} >25\,\mathrm{{GeV}}$ and $|\eta |<4.7$. The cross section is measured to be $\sigma ( {{\mathrm {p}\mathrm {p}}} \rightarrow {\mathrm {W}} (\ell \nu )$+${\mathrm{{b}}\mathrm{{\overline{b}}}})= 0.64 \pm 0.03{\,\mathrm{{(stat)}}} \pm 0.10{\,\mathrm{{(syst)}}} \pm 0.06{\,\mathrm{{(theo)}}} \pm 0.02{\,\mathrm{{(lumi)}}}\,{\text {pb}}$, in agreement with standard model predictions.

## Introduction

The measurement of $\mathrm {W}$ or $\mathrm {Z}$ boson production in association with b quarks in proton–proton collisions provides important input for refinement of calculations in perturbative quantum chromodynamics and is also relevant for searches and measurements. In particular, these processes constitute a background to the experimental measurement of a standard model (SM) Higgs boson in which the Higgs boson decays into a ${\mathrm{b} \mathrm{{\overline{b}}}}$ pair in association with a vector boson. The discovery by the ATLAS and CMS Collaborations at the CERN LHC of a neutral boson with a mass of about $125\,\mathrm{{GeV}}$ [1–4] motivates further studies to establish the nature of the boson and determine its coupling to bottom quarks. Furthermore, different models based on extensions of the Higgs sector are being compared with LHC data using final states composed of leptons and b jets. In this context, a better understanding of the b hadron production mechanism and the kinematic properties of associated jets is required to refine the background predictions and increase the sensitivity to new physics. Throughout this paper, hadronic showers originating from bottom or anti-bottom quarks are referred to as b jets, and b-tagged jets are the reconstructed objects either in simulation or data that have been identified as such.

The production of $\mathrm {W}$ [5, 6] or $\mathrm {Z}$ [7–11] bosons in association with b jets has been measured at the LHC using pp collisions at $\sqrt{s}=7{\,\mathrm{{TeV}}}$ using data samples corresponding to an integrated luminosity of up to 5$\,\text {fb}^\text {-1}$, and at the Fermilab Tevatron [12, 13] using proton–antiproton collisions at $\sqrt{s}=1.96{\,\mathrm{{TeV}}}$. This analysis extends previous measurements of the ${\mathrm {W}}+{\mathrm{{b}}\mathrm{{\overline{b}}}}$ production cross section [5] and uses data at $\sqrt{s}=8{\,\mathrm{{TeV}}}$ collected with the CMS detector, corresponding to an integrated luminosity of 19.8$\,\text {fb}^\text {-1}$  [14]. Whereas the previous CMS analysis used only the muon decay channel, this analysis uses both muon and electron decay modes.

## CMS detector

The central feature of the CMS apparatus is a superconducting solenoid of 6 m internal diameter, providing a magnetic field of 3.8 T. Within the solenoid volume are a silicon pixel and strip tracker, a lead tungstate crystal electromagnetic calorimeter (ECAL), and a brass and scintillator hadron calorimeter (HCAL), each composed of a barrel and two endcap sections. Muons are measured in gas-ionization detectors embedded in the steel flux-return yoke outside the solenoid. Extensive forward calorimetry complements the coverage provided by the barrel and endcap detectors. A more detailed description of the CMS detector, together with a definition of the coordinate system used and the relevant kinematic variables, can be found in Ref. [15].

## Event selection and reconstruction

The $\mathrm {W}\rightarrow \mathrm {\mu }\nu _\mathrm {\mu }$ ($\mathrm {W}\rightarrow \mathrm {e}\nu _\mathrm {e}$) events are selected using single-muon (single-electron) triggers that require a loosely isolated muon (electron) with transverse momentum $p_{\mathrm {T}} >24\,(27)\,\mathrm{{GeV}}$ and pseudorapidity $|\eta |<2.1\,(2.5)$.

Individual particles emerging from each collision are reconstructed with the particle-flow (PF) technique [16, 17]. This approach uses the information from all subdetectors to identify and reconstruct individual particle candidates in the event, classifying them into mutually exclusive categories: charged and neutral hadrons, photons, electrons, and muons.

Muons are reconstructed by combining the information from the tracker and the muon spectrometer [18, 19]. Electrons are reconstructed by combining the information from the tracker and the calorimeter [20]. Both the muon and the electron candidates are required to have $p_{\mathrm {T}} >30\,\mathrm{{GeV}}$ and $|\eta |<2.1$ to ensure that the triggers are fully efficient. They are also required to originate from the primary vertex of the event, chosen as the vertex with the highest $\sum p_{\mathrm {T}} ^2$ of the charged particles associated with it. Furthermore, the leptons must be isolated, where the isolation variable is defined as

1 $$\begin{aligned} I= & {} \frac{1}{p_{\mathrm {T}} ^\ell }\left[ \sum p_{\mathrm {T}} ^{\text {charged}}\right. \nonumber \\&\left. +{\max }\Bigl (0,\textstyle {\sum }p_{\mathrm {T}} ^{\gamma }+\textstyle {\sum }E_{\mathrm {T}} ^{\text {neutral}}-0.5\,\textstyle {\sum } p_{\mathrm {T}} ^{\mathrm {PU}}\Bigr )\right] , \end{aligned}$$

with the sums running over PF candidates in a cone of size $\varDelta R < 0.4\,(0.3)$ around the muon (electron) direction, where $\varDelta R = \sqrt{\smash [b]{ (\varDelta \eta )^2 + (\varDelta \phi )^2}}$, and $\phi$ is the azimuthal angle in radians. The first three sums are over charged hadron candidates associated with the primary vertex, photon candidates, and neutral hadron candidates respectively. The definition of the isolation includes a correction for additional pp interactions, referred to as pileup, which is proportional to the scalar $p_{\mathrm {T}}$ sum of charged particles not associated with the primary vertex in the isolation cone ($\sum p_{\mathrm {T}} ^{\mathrm {PU}}$). The selected muons (electrons) are required to have $I < 0.12\,(0.10)$.

Missing transverse momentum in the event, ${\vec p}_{\mathrm {T}}^\mathrm{{miss}}$, is defined as the negative vector sum of the $\vec {p_{\mathrm {T}}}$ of all PF candidates in the event. It is combined with the $\vec {p_{\mathrm {T}}}$ of a muon or electron passing the identification and isolation requirements to compute the transverse mass, $M_\mathrm {T}$, of the $\mathrm {W}$ boson candidate. The $M_\mathrm {T}$ variable is a natural discriminator against non-$\mathrm {W}$ final states, such as quantum chromodynamics (QCD) multijet events, that have a lepton candidate and ${\vec p}_{\mathrm {T}}^\mathrm{{miss}}$, but a relatively low value of $M_\mathrm {T}$. The result for ${\vec p}_{\mathrm {T}}^\mathrm{{miss}}$ is corrected for noise in the ECAL and HCAL using the method described in Ref. [21]. Corrections to minimize the effect of the pileup are also included [22].

Jets are constructed using the anti-$k_{\mathrm {T}}$ clustering algorithm [23] with a radius parameter of 0.5, as implemented in the FastJet package [24, 25]. Jet clustering is performed using individual particle candidates reconstructed with the PF technique. Jets are required to pass identification criteria that eliminate jets originating from noisy channels in the HCAL [26]. Jets from pileup interactions are rejected by requiring that the jets originate at the primary interaction vertex. Small corrections to the relative and absolute jet energy calibrations of the detector are applied as a function of the $p_{\mathrm {T}}$ and $\eta$ of the jet [27].

The combined secondary vertex (CSV) b tagging algorithm [28, 29] exploits the long lifetime and relatively large mass of b hadrons to provide b jet identification. The CSV algorithm combines information about impact parameter significance, secondary vertex kinematic properties, and jet kinematic properties in a likelihood-ratio discriminator. The identification of b jets (b tagging) is made by imposing a minimum threshold on the CSV discriminator value. In this analysis, b-tagged jets are required to pass a threshold with an efficiency of 40% in the signal phase space and a misidentification probability of 0.1% (1%) for light (charm) jets. Jets are corrected for the difference in efficiency between data and simulation using scale factors dependent on the $p_{\mathrm {T}}$ of the jet.

## Simulated samples

After all selection requirements detailed in Sect. 5 are applied, the contributing background processes to the overall yield are the associated production of a massive vector boson and jets ($\mathrm {V}$+jets where V = $\mathrm {W}$ or $\mathrm {Z}$), as well as production of diboson ($\mathrm {W^+}{}\mathrm {W^-}$, $\mathrm {W}{}\mathrm {Z}$, $\mathrm {Z}{}\mathrm {Z}$), $\mathrm{{t}}\mathrm{{\overline{t}}}$, single top quark, $\gamma$+jets, and QCD multijet events. These background contributions are estimated from simulation, except for the QCD background, which is estimated from data as described in Sect. 5.

Simulated samples of $\mathrm {V}$+jets, $\gamma$+jets and $\mathrm{{t}\mathrm{{\overline{t}}}}+\mathrm{{jets}}$ are generated at tree-level with MadGraph  5.1 [30, 31] using the CTEQ6L [32] parton distribution function (PDF) set. These samples are interfaced with pythia  6.4 [33] for hadronization using the Z2* tune for the underlying event. The most recent pythia Z2* tune is derived from the Z1 tune [34], which uses the CTEQ5L PDF set, whereas Z2* adopts CTEQ6L [32]. The $k_{\mathrm {T}}$-MLM [35, 36] matching scheme is used. For the signal distributions, the shapes are taken from a dedicated high-statistics generated sample of exclusive ${\mathrm {W} + \mathrm{{b}}\mathrm{{\overline{b}}}}$. The normalization is obtained from the ${\mathrm {W} + \mathrm{{b}}\mathrm{{\overline{b}}}}$ component of an inclusive $\mathrm {W}$+jets sample by separating the $\mathrm {W}$+jets simulated sample into three subsamples labeled as ${\mathrm {W} + \mathrm{{b}}\mathrm{{\overline{b}}}}$, $\mathrm {W}$+$\mathrm {c} \mathrm {\overline{c}}$ , and $\mathrm {W}$+$\mathrm {u}$ $\mathrm {d}$ $\mathrm {s}$ $\mathrm {c}$ $\mathrm {g}$ , which are defined below. If an event contains a bottom jet from the matrix element or parton shower, it is categorized as ${\mathrm {W} + \mathrm{{b}}\mathrm{{\overline{b}}}}$. A bottom quark at generator level requires the presence of a bottom hadron within a cone of radius $\varDelta R=0.4$ with respect to the jet axis. The jets are constructed using generator-level information using all stable particles in the event, excluding neutrinos. Jets with a distance smaller than $\varDelta R = 0.5$ with respect to a lepton are removed from the event. If an event does not contain any b jet, but an even, nonzero number of charm jets, again from the matrix element or parton shower, it is categorized as $\mathrm {W}$+$\mathrm {c} \mathrm {\overline{c}}$ . The remaining events are categorized as $\mathrm {W}$+$\mathrm {u}$ $\mathrm {d}$ $\mathrm {s}$ $\mathrm {c}$ $\mathrm {g}$ . The energy of the selected leptons at the generator level is corrected for final-state radiation by summing the four-momenta of all the photons generated within a cone of radius $\varDelta R = 0.1$ around the lepton. Leptons that do not originate from the primary vertex are not considered for selection.

Single top quark event samples are generated at next-to-leading order (NLO) with powheg  2.0 [37–40] using the CTEQ6M PDF set. Hadronization is performed using pythia  6.4 with the Z2* tune. Diboson samples are generated and hadronized with pythia  6.4 at leading order (LO) using the CTEQ6L PDF set and the Z2* tune.

The cross sections for the $\mathrm {V}$+jets processes are normalized using the predictions for inclusive $\mathrm {W}$ and $\mathrm {Z}$  boson production from fewz  3.1 [41, 42] evaluated at next-to-next-to-leading order (NNLO). The cross section for $\gamma$+jets is evaluated at LO using MadGraph with the CTEQ6L PDF set. Single top quark and diboson production cross sections are normalized to the NLO cross section predictions from $\textsc {mcfm} ~7.0$ [43, 44] using the MSTW2008 NLO PDF set [45]. The $\mathrm{{t}}\mathrm{{\overline{t}}}$ cross section used is ${241.5\pm 8.5\,{\text {pb}}}$, and was determined from data collected by the ATLAS and CMS experiments [46–48] at the LHC at $\sqrt{s}=8{\,\mathrm{{TeV}}}$.

For all simulated processes, the detector response is simulated using a detailed description of the CMS detector based on Geant4  [49]. The reconstruction of simulated events is performed with the same algorithms used for the data.

Events induced by additional simultaneous pp interactions are simulated using events generated with pythia  6. During the 2012 data taking, the average pileup rate was 21 interactions per bunch crossing; the simulated number of pileup interactions has been reweighted to match this distribution in the data.

## Analysis strategy

The ${\mathrm {W} + \mathrm{{b}}\mathrm{{\overline{b}}}}$ yield is estimated using a binned maximum-likelihood fit to the $M_\mathrm {T}$ distribution in the signal event sample. With the exception of the multijet processes, the distributions and normalizations of all background contributions in the fit are taken from simulation. Consequently, it is important to verify that the simulation describes the data.

The dominant background in the signal event sample arises from the $\mathrm{{t}}\mathrm{{\overline{t}}}$ process. Therefore, the data and simulation are compared in two $\mathrm{{t}}\mathrm{{\overline{t}}}$-dominated control samples: one characterized by a pair of opposite flavor leptons ($\mathrm{{t}}\mathrm{{\overline{t}}}$-multilepton), and the other by the presence of three or more jets ($\mathrm{{t}}\mathrm{{\overline{t}}}$-multijet). The simulation is reweighted to describe the data in the control regions and then is used to predict the $M_\mathrm {T}$ distributions in the signal region.

The signal region contains a muon (electron) with $p_{\mathrm {T}} > 30\,\mathrm{{GeV}}$, $|\eta | < 2.1$, and satisfying $I < 0.12\,(0.10)$. Exactly two b-tagged jets with $p_{\mathrm {T}} > 25\,\mathrm{{GeV}}$ and $|\eta | < 2.4$ are also required. Events with additional leptons with $p_{\mathrm {T}} > 10\,\mathrm{{GeV}}$ and $|\eta | < 2.4$ or a third jet with $p_{\mathrm {T}} > 25\,\mathrm{{GeV}}$ and $|\eta | < 4.7$ are rejected. The $\mathrm{{t}}\mathrm{{\overline{t}}}$-multijet sample is obtained using the same selection criteria as for the signal event sample, but requiring at least three jets in the event with $p_{\mathrm {T}} >25\,\mathrm{{GeV}}$ and $|\eta |<2.4$ instead of vetoing events that have more than two jets. The $\mathrm{{t}}\mathrm{{\overline{t}}}$-multilepton sample uses similar selection criteria as the signal event sample; however, the lepton requirement is modified. The event must contain two isolated leptons of different flavor, each with $p_{\mathrm {T}} >30\,\mathrm{{GeV}}$ and $|\eta |<2.1$. In the $\mathrm{{t}}\mathrm{{\overline{t}}}$-multilepton sample, the $M_\mathrm {T}$ variable is calculated with respect to the electron in the electron channel and the muon in the muon channel.

The QCD background distributions in the $M_\mathrm {T}$ variable are estimated from data using event samples that pass all signal requirements, but requiring the muon (electron) is not isolated, $I > 0.20\,(0.15)$. The resulting distributions are corrected for the presence of all other backgrounds, as estimated from simulation. Their contribution is less than 1% of the QCD background rate. The QCD background normalization is adjusted in order to describe the number of data events at $M_\mathrm {T} <20\,\mathrm{{GeV}}$, after subtracting the non-QCD backgrounds obtained from simulation.

In the fiducial regions used in this analysis, no correlation is observed between *I* and $M_\mathrm {T}$ in multijet events simulated with pythia 6, so the use of an inverted isolation requirement to obtain the QCD background distribution is possible. However, this is not the case for the $\varDelta R$ distance between the two b-tagged jets, $\varDelta R({\mathrm{{b}},\mathrm{{\overline{b}}}})$, or the lepton $p_{\mathrm {T}}$. The shape of the QCD distribution for these variables is therefore taken from an $M_\mathrm {T} <30$ $\,\mathrm{{GeV}}$ sideband and validated against QCD multijet simulation. The normalization of the QCD background in these variables is set to the final normalization resulting from the fit to the $M_\mathrm {T}$ variable, which was derived using the inverted isolation requirement.

The normalizations and distributions of the simulated backgrounds are allowed to vary in the fit within the uncertainties listed in Table 1 as described in Sect. 6. The uncorrelated normalization uncertainties are uncertainties in the cross section of the given sample.

Two major parameters in the simulations significantly affect the normalization of the simulated distributions: the b tagging efficiency and the jet energy scale (JES). The control samples as well as the signal event samples show similar sensitivity to the b tagging efficiency, and its adjustment affects all the regions in a correlated manner. Because $\mathrm{{t}}\mathrm{{\overline{t}}}$ production may have more than two jets in the final state, the rejection of events with a third jet makes it sensitive to JES. The effect on the leading jets is moderate, but JES variations lead to significant migration of jets into and out of the veto region. The $\mathrm{{t}}\mathrm{{\overline{t}}}$-multijet sample, since it has no veto on a third jet, is less sensitive to JES variations than the $\mathrm{{t}}\mathrm{{\overline{t}}}$-multilepton sample. The variation in the JES changes the ${\mathrm {W} + \mathrm{{b}}\mathrm{{\overline{b}}}}$ yield in the signal region by less than 1%.

The fit procedure consists of three consecutive steps in which the simulated distributions in two control samples and the event sample are fit to data using the $M_\mathrm {T}$ variable, which is chosen because it has a well-known shape for $\mathrm {W}$+jets production that allows for reliable signal extraction. First, the fit is performed using the $\mathrm{{t}}\mathrm{{\overline{t}}}$-multijet sample. It results in a correction of the b tagging efficiency, measured separately in the muon and electron channels and then combined. The simulation is corrected using this result and the corrected simulated samples are fit to the data in the $\mathrm{{t}}\mathrm{{\overline{t}}}$-multilepton sample. The result of the second step is used to adjust JES and as a result of this procedure, the simulation is expected to better describe the $\mathrm{{t}}\mathrm{{\overline{t}}}$ contribution. The final step is to extract the number of ${\mathrm {W} + \mathrm{{b}}\mathrm{{\overline{b}}}}$ events from the fit to $M_\mathrm {T}$ in the signal event sample.

Similar results can be obtained by performing a simultaneous fit of the signal and the two control regions. We find that the b tagging efficiency correction and JES correction have opposite effects on the distributions and thus compensate for each other in a simultaneous fit, reducing its precision. Separating these effects in steps provides better understanding of underlying uncertainties and therefore more precise results.

## Systematic uncertainties

The main sources of the systematic uncertainties are listed in Table 1. The size of the variation is shown for each source, together with its effect on the measured cross section. These are included in the fit. Some of the uncertainties affect only the normalization in the respective contributions. These include the uncertainties in the theoretical cross section for a given sample, which are uncorrelated between samples and are included as log-normal constraints on the rate. The uncertainty due to the b tagging efficiency and the uncertainty due to the JES are observed to only affect the normalizations of the samples in the $M_\mathrm {T}$ variable. The uncertainties that affect both the normalization and the shape of the $M_\mathrm {T}$ distributions are listed in the table under “Shape” and are incorporated into the fit via binned distributions, which are obtained by varying the source of the given uncertainty and reprocessing the simulated sample. Such uncertainties in the template are interpolated quadratically.

As a conservative estimate of the uncertainty in QCD multijet background, a 50% uncertainty has been considered. This results in an uncertainty of 2–3% in the measured cross section. The b tagging efficiency and JES rescaling uncertainties are taken from their respective fits. The renormalization and factorization scales respectively are set at $\mu _{\mathrm {R}}=\mu _{\mathrm {F}}=m_\mathrm {W}$, and the uncertainties on this choice are estimated from the change in acceptance found by varying $\mu _{\mathrm {R}}$ and $\mu _{\mathrm {F}}$ up and down by a factor of two. The PDF uncertainties are estimated from the change in acceptance found by varying the PDF set following the LHAPDF/PDF4LHC prescription [50–53], considering PDF sets from the CTEQ, MSTW, NNPDF, and HERA Collaborations.

**Table 1:** The main sources of systematic uncertainty in the ${\mathrm {W} + \mathrm{{b}}\mathrm{{\overline{b}}}}$ signal event sample. The column labeled “Variation” indicates the bounds on the normalization change of a given sample due to a variation of the uncertainty by one standard deviation. The last column indicates the contribution of the given systematic to the overall uncertainty in the measured cross section. The uncertainty labeled “b tag eff rescaling” is the uncertainty associated with the rescaling of the b tagging efficiency. UES refers to the scale of energy deposits not clustered into jets, and MES and EES refer to the muon and electron energy scales. The uncertainty labeled as “Id/Iso/Trg” is the uncertainty associated with the efficiency of the lepton identification, isolation, and trigger. The uncertainties in the integrated luminosity [14] and in the acceptance due to PDF uncertainties and scale choices are not included in the fit, and are treated separately

				Effect on the
				measured
		Uncertainty	Variation	cross section
Uncorrelated	Normalization	$\mathrm{{t}}\mathrm{{\overline{t}}}$	3.5%	3.8%
		Single top	5.4%	2.5%
		$\mathrm {W}$+$\mathrm {u}$ $\mathrm {d}$ $\mathrm {s}$ $\mathrm {c}$ $\mathrm {g}$	13.2%	${<}2$%
		$\mathrm {W}$+$\mathrm {c} \mathrm {\overline{c}}$	13.2%	${<}2$%
		Diboson	8.1%	${<}2$%
		Drell–Yan	7.9%	${<}2$%
		$\gamma$+jets	10.0%	${<}2$%
		QCD	50%	2–3%
Correlated	Normalization	b tag eff rescaling	8.4%	9.2%
		JES rescaling	0–6%	3.8%
		UES	0–3%	${<}2$%
		MES	0–3%	${<}2$%
		EES	0–3%	${<}2$%
		Id/Iso/Trg	0–4%	${<}2$%
		Luminosity	2.6%	
		Scales ($\mu _{\mathrm {R}}$,$\mu _{\mathrm {F}}$)	10%	
		PDF choice	1%	

## Results

**Fig. 1:**
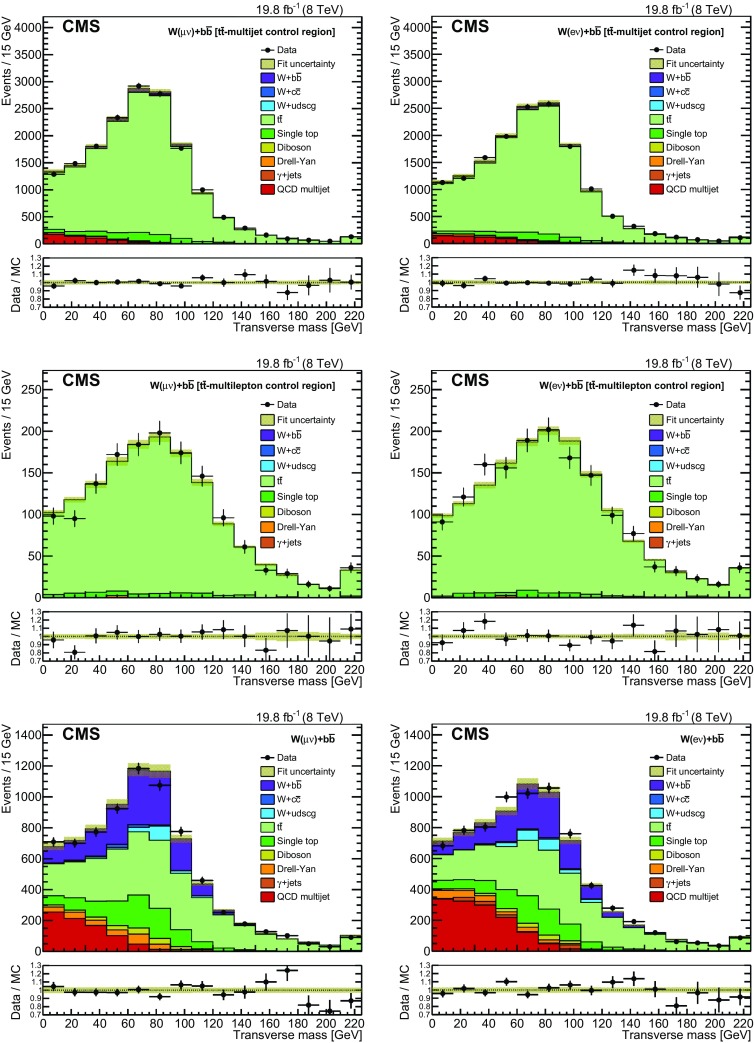
The transverse mass distributions (*upper*) in the $\mathrm{{t}}\mathrm{{\overline{t}}}$-multijet phase space after fitting to obtain the b tagging efficiency rescale factors, (*middle*) in the $\mathrm{{t}}\mathrm{{\overline{t}}}$-multilepton sample after fitting to find the appropriate JES, and (*lower*) in the ${\mathrm {W} + \mathrm{{b}}\mathrm{{\overline{b}}}}$ signal sample after fitting simultaneously muon and electron decay channels. The lepton channels are shown separately with the muon sample on the *left* and the electron sample on the *right*. The last bin contains overflow events. The *shaded area* represents the total uncertainty in the simulation after the fit

The fit in the $\mathrm{{t}}\mathrm{{\overline{t}}}$-multijet sample is used to obtain b tagging efficiency rescaling factors separately for the muon and electron channels in order to better describe the b tagging efficiency in the simulation as described in Sect. 5. The results of the fit are presented in the two plots at the top of Fig. 1. The central values of the b tagging efficiency rescaling factors, $1.12 \pm 0.08$ (muon channel) and $1.16 \pm 0.08$ (electron channel), are averaged to $1.14 \pm 0.08$ with the combined uncertainty, dominated by systematics, taken as the maximum of the uncertainties for the individual lepton channels. The simulation is reweighted accordingly for the next fit, and the uncertainty in this fit sets the one standard deviation bound on the b tagging efficiency rescaling factor in subsequent fits.

A fit to the $\mathrm{{t}}\mathrm{{\overline{t}}}$-multilepton sample adjusts the JES, as described in Sect. 5. As a result, the simulated $M_\mathrm {T}$ distributions change normalization. The best fit results in changing the normalization by approximately 3.4% from its central value, which corresponds to 1.3 standard deviations in JES. The middle plots in Fig. 1 show the results of the fits in the $\mathrm{{t}}\mathrm{{\overline{t}}}$-multilepton sample for the muon (left) and the electron (right) channels. The JES is therefore shifted by 1.3 standard deviations in the simulation with the uncertainty taken from the fit. Thus the simulation is tuned to describe the $\mathrm{{t}}\mathrm{{\overline{t}}}$ control samples and is used to extract the signal yield in the signal region.

The results of the fit in the ${\mathrm {W} + \mathrm{{b}}\mathrm{{\overline{b}}}}$ signal region are shown in the bottom of Fig. 1. All background contributions are allowed to vary in the fit within their uncertainties, while the ${\mathrm {W} + \mathrm{{b}}\mathrm{{\overline{b}}}}$ normalization remains a free parameter of the fit. The correlations across all simulated samples are taken into account as shown in Table 1. Based on the fits the number of events of each type in the signal event sample is given in Table 2. Events coming from the production of a Higgs boson in association with a vector boson constitute a negligible fraction of the overall event yield and are not considered.

**Fig. 2:**
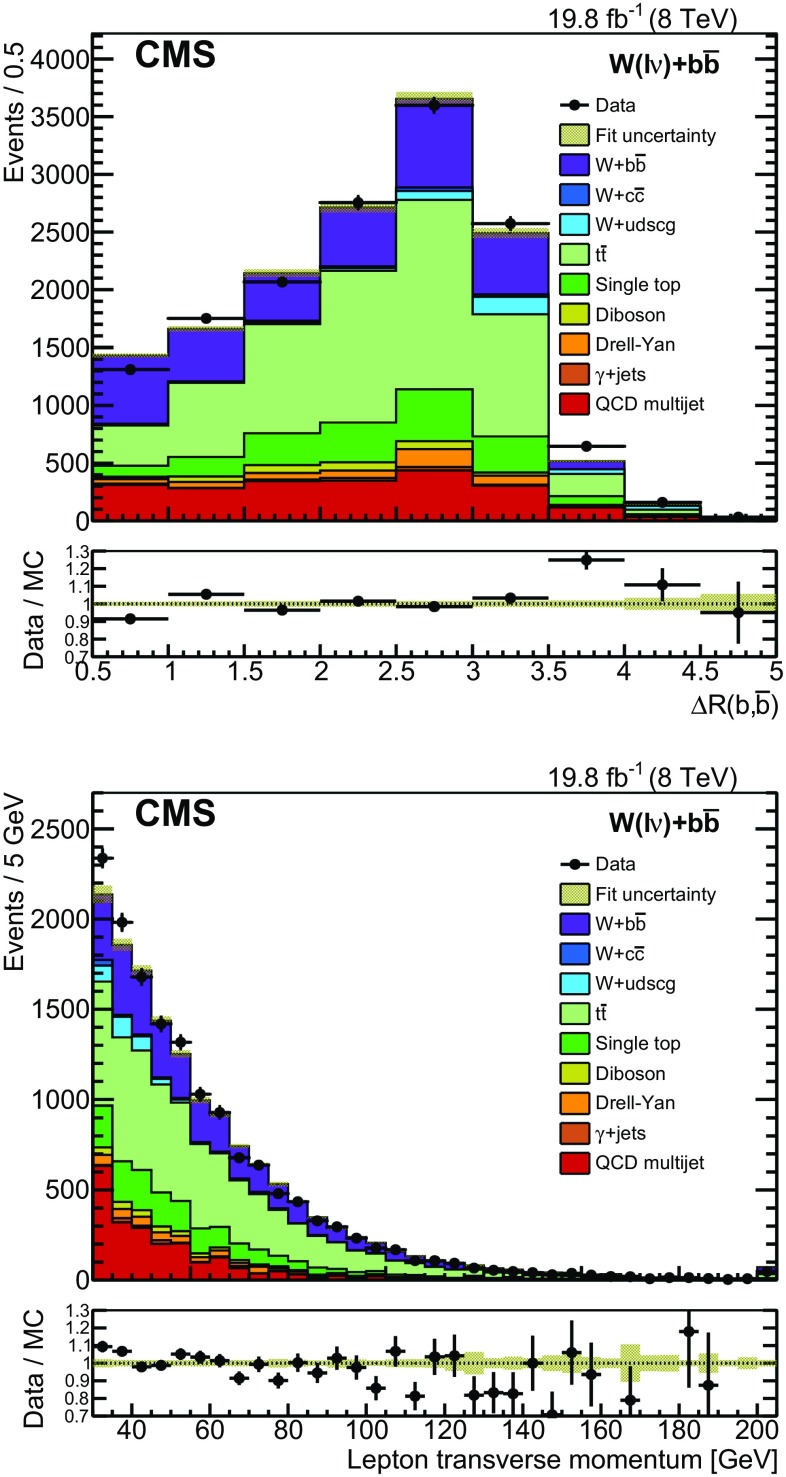
Distributions of $\varDelta R({\mathrm{{b}},\mathrm{{\overline{b}}}})$ and $p_{\mathrm {T}} ^\ell$ after applying the results from the fits to the simulation. The QCD background shape is taken from an $M_\mathrm {T} <30\,\mathrm{{GeV}}$ sideband and the muon and electron channels have been combined in these distributions. The last bin contains overflow events and the *shaded area* represents the total uncertainty in the simulation after the fit

Distributions for variables other than those being directly fit are also produced by applying the results from the three fits to the simulated samples. Distributions of $\varDelta R({\mathrm{{b}},\mathrm{{\overline{b}}}})$ and $p_{\mathrm {T}} ^\ell$ combining both lepton flavors are presented in Fig. 2. The angular separation between the b jets is seen to be well modeled, and the $p_{\mathrm {T}} ^\ell$ distribution shows an agreement within $10\%$ for $p_{\mathrm {T}} ^\ell < 100$ GeV, with a slightly falling trend in the ratio of data and simulation.

**Table 2:** Initial and final yields obtained in the ${\mathrm {W} + \mathrm{{b}}\mathrm{{\overline{b}}}}$ signal region. The uncertainties in the signal strength represent the total uncertainty of the fit

	Muon		Electron	
	Initial	Fitted	Initial	Fitted
Data	7432		7357	
${\mathrm {W} + \mathrm{{b}}\mathrm{{\overline{b}}}}$	1323	1712	1121	1456
$\mathrm {W}$+$\mathrm {c} \mathrm {\overline{c}}$	60	61	36	37
$\mathrm {W}$+$\mathrm {u}$ $\mathrm {d}$ $\mathrm {s}$ $\mathrm {c}$ $\mathrm {g}$	182	179	220	217
$\mathrm{{t}}\mathrm{{\overline{t}}}$	3049	3296	2640	2864
Single top	958	1008	820	865
Drell–Yan	261	265	220	224
Diboson	175	181	139	144
$\gamma +$jets	—	—	98	105
QCD	1109	803	1654	1373
Total MC	7116	7505	6948	7284
Signal strength	$1.21 \pm 0.19$		$1.37 \pm 0.23$	
Combined	$1.26 \pm 0.17$			

The cross section is calculated as

$$\begin{aligned} \sigma (\mathrm {p}\mathrm {p}\rightarrow \mathrm {W}(\ell \nu )+{\mathrm{{b}}\mathrm{{\overline{b}}}}) =&\frac{N^{\text {data}}_{\text {reconstructed}}}{A\,\epsilon \, \mathcal {L}} \\ =&\frac{N^{\text {data}}_{\text {reconstructed}}}{(N^{\mathrm {MC}}_{\text {reconstructed}}/N^{\mathrm {MC}}_{\text {generated}})\, \mathcal {L}} = \alpha \sigma _{\text {gen}} \end{aligned}$$

where $\mathcal {L}$ is the integrated luminosity, $N^{\text {data}}_{\text {reconstructed}}$ is the number of observed signal events, $N^{\mathrm {MC}}_{\text {reconstructed}}$ is the number of expected signal events from simulation reconstructed in the fiducial region, $N^{\mathrm {MC}}_{\text {generated}}$ is the number of generated events in the fiducial region, *A* and $\epsilon$ are the acceptance and efficiency, $\alpha$ is the measured signal strength in the given lepton channel, and $\sigma _{\text {gen}}$ is the simulated fiducial cross section of the signal sample. The signal strength is the scale factor in the ${\mathrm {W} + \mathrm{{b}}\mathrm{{\overline{b}}}}$ cross section predicted by the fit, after factoring out contributions to the overall change in normalization due to systematic effects which are correlated across samples. In this analysis, the fiducial cross section is calculated as follows: MadGraph is used to compute the ${\mathrm {W} + \mathrm{{b}}\mathrm{{\overline{b}}}}$ cross section with fiducial selections applied. Then a k-factor for inclusive W production is applied that is obtained from the ratio of the inclusive W cross section calculated with fewz  3.1 (at NNLO using the five-flavour CTEQ6M PDF set) and to that with MadGraph. The product $A\,\epsilon$ is 10 to 15% and results from the combined effects of the efficiency of the lepton identification requirements (80%) and b tagging efficiency (40% per jet) and has an uncertainty of 10%, arising from scale and PDF choices as indicated in the bottom of Table 1.

The ${\mathrm {W} + \mathrm{{b}}\mathrm{{\overline{b}}}}$ cross section is measured within a fiducial volume, which is defined by requiring leptons with $p_{\mathrm {T}} > 30\,\mathrm{{GeV}}$ and $|\eta |<2.1$ and exactly two b-tagged jets of $p_{\mathrm {T}} > 25\,\mathrm{{GeV}}$ and $|\eta |<2.4$. The measured cross sections are presented in Table 3. The combination of the muon and electron measurements is done using a simultaneous fit to both channels, taking into account correlations across samples.

**Table 3:** Measured cross sections in the muon, electron, and combined lepton channels. The systematic uncertainty (syst) includes the contributions from all rows in Table 1 that have an entry in the “Variation” column, and the theoretical uncertainty (theo) includes the combination of the uncertainties associated with the choice of $\mu _{\mathrm {R}}$, $\mu _{\mathrm {F}}$, and PDF

Channel	$\sigma (\mathrm {p}\mathrm {p}\rightarrow \mathrm {W}(\ell \nu )+{\mathrm{{b}}\mathrm{{\overline{b}}}})\,\mathrm{{pb}}$
Combined	$0.64 \pm 0.03{\,\mathrm{{(stat)}}}\pm 0.10{\,\mathrm{{(syst)}}}\pm 0.06{\,\mathrm{{(theo)}}}\pm 0.02{\,\mathrm{{(lumi)}}}$
Muon	$0.62 \pm 0.04{\,\mathrm{{(stat)}}}\pm 0.11{\,\mathrm{{(syst)}}}\pm 0.06{\,\mathrm{{(theo)}}}\pm 0.02{\,\mathrm{{(lumi)}}}$
Electron	$0.70 \pm 0.05{\,\mathrm{{(stat)}}}\pm 0.15{\,\mathrm{{(syst)}}}\pm 0.07{\,\mathrm{{(theo)}}}\pm 0.02{\,\mathrm{{(lumi)}}}$

The measured cross sections are compared to theoretical predictions from mcfm  7.0 [43, 44] with the MSTW2008 PDF set, as well as from MadGraph  5 interfaced with pythia  6 in the four- and five-flavour schemes and MadGraph  5 with pythia  8 [54] in the four-flavour scheme. In the four- and five-flavour approaches, the four and five lightest quark flavours are used in the proton PDF sets. In the five-flavour scheme, the PDF set CTEQ6L is used and interfaced with pythia 6 using the Z2* tune. The two four-flavour samples are produced using an NNLO PDF set interfaced with pythia version 6 using the CTEQ6L tune in one sample, and version 8 using the CUETP8M1 tune [55] in the other.

Comparisons between the results of calculations performed under different assumptions provide important feedback on the validity of the techniques employed. Differences in predictions arising from the modelling of b quarks as massive or massless are possible, as are variations in predictions arising from the use of different showering packages (pythia 6 vs. pythia 8) or matrix element generators (MadGraph vs. mcfm  7.0). In the phase space explored here, these predictions are all very close in their central value and agree with each other well within their respective uncertainties.

The mcfm  7.0 cross section calculation is performed at the level of parton jets and thus requires a hadronization correction. The multiplicative hadronization correction factor $0.81\pm 0.07$ is calculated using the MadGraph + pythia  6 sample and agrees well with the factor $0.84\pm 0.03$ calculated in the 7 TeV Z+b analysis [8]. The correction factor is obtained for jets computed excluding neutrinos from the particle list because such jets are closer in kinematics to particle jets at the detector level. The uncertainty reflects both the limited statistics of the MadGraph + pythia  6 sample as well as a comparison with the MadGraph + pythia  8 sample.

The mcfm  7.0 and four-flavour MadGraph predictions do not take into account ${\mathrm {W} + \mathrm{{b}}\mathrm{{\overline{b}}}}$ production where the ${\mathrm{{b}}\mathrm{{\overline{b}}}}$ system is produced in a different partonic level interaction than the one which produced the $\mathrm {W}$ boson, albeit in the same collision. Simulations of MadGraph + pythia events that include double parton interactions (DPI) reproduce the $\mathrm {W}$+jets data [56]. Therefore a MadGraph + pythia  8 sample of a $\mathrm {W}$ boson produced in association with a ${\mathrm{{b}}\mathrm{{\overline{b}}}}$ pair coming from DPI is generated to study the effect on the fiducial cross section. Using this dedicated sample, an additive correction $\sigma _{\mathrm {DPI}}$ is estimated to be $0.06\pm 0.06\,{\text {pb}}$, where the uncertainty is conservatively assigned to be 100% of the value.

**Fig. 3:**
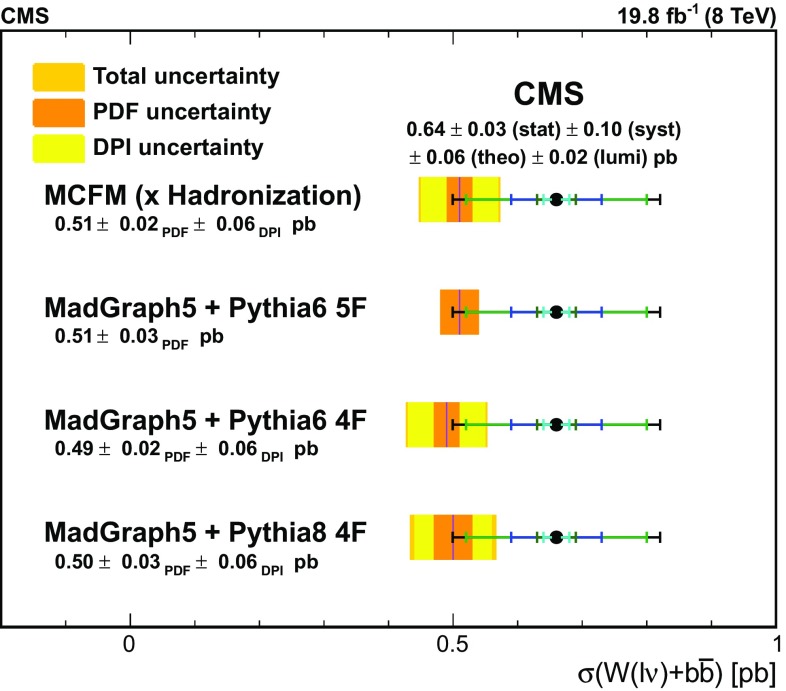
Comparison between the measured ${{\mathrm {W}}(\ell \nu )}+{\mathrm{{b}}\mathrm{{\overline{b}}}}$ cross section and various QCD predictions. The *orange band* indicates the uncertainty in the given sample associated with PDF choice and the *yellow band* represents the uncertainty associated with DPI. The labels 4F and 5F refer to the four- and five-flavour PDF schemes. In the case of the MadGraph + pythia 6 (5F) sample, the effects of DPI are already included in the generated samples so the DPI correction is not needed. The measured cross section is also shown with the total uncertainty in black and the luminosity, statistical, theoretical, and systematic uncertainties indicated

The resulting cross section predictions in the fiducial phase space at the hadron level, including the estimated hadronization and DPI corrections as needed, are compared in Fig. 3 with the measured value. Within one standard deviation the predictions agree with the measured cross section.

## Summary

The cross section for the production of a W boson in association with two b jets was measured using a sample of proton–proton collisions at $\sqrt{s} = 8{\,\mathrm{{TeV}}}$ collected by the CMS experiment. The data sample corresponds to an integrated luminosity of 19.8$\,\text {fb}^\text {-1}$. The W bosons were reconstructed via their leptonic decays, $\mathrm {W}\rightarrow \ell \nu$, where $\ell =\mu$ or $\mathrm {e}$. The fiducial region studied contains exactly one lepton with transverse momentum $p_{\mathrm {T}} ^{\ell }>30\,\mathrm{{GeV}}$ and pseudorapidity $|\eta ^{\ell } |<2.1$, with exactly two b jets with $p_{\mathrm {T}} >25\,\mathrm{{GeV}}$ and $|\eta |<2.4$ and no other jets with $p_{\mathrm {T}} >25\,\mathrm{{GeV}}$ and $|\eta |<4.7$. The cross section is $\sigma ( {{\mathrm {p}\mathrm {p}}} \rightarrow {\mathrm {W}} (\ell \nu )+{\mathrm{{b}}\mathrm{{\overline{b}}}})= 0.64 \pm 0.03{\,\mathrm{{(stat)}}}\pm 0.10{\,\mathrm{{(syst)}}}\pm 0.06{\,\mathrm{{(theo)}}}\pm 0.02{\,\mathrm{{(lumi)}}}\,{\text {pb}}$, in agreement with standard model predictions.
